# Cognitive Effects of Lurasidone and Cariprazine: A Mini Systematic Review

**DOI:** 10.2174/1570159X21666230727140843

**Published:** 2023-09-25

**Authors:** Miriam Olivola, Nicola Bassetti, Serena Parente, Vincenzo Arienti, Serena Chiara Civardi, Pietro Alessandro Topa, Natascia Brondino

**Affiliations:** 1 Department of Brain and Behavioral Sciences, Università di Pavia, Pavia, 27100, Italy;; 2 Department of Mental Health and Addiction, ASST Pavia, Pavia, Italy

**Keywords:** Antipsychotics, lurasidone, cariprazine, 5HT7, D3, cognition, psychosis, affective disorder

## Abstract

Cognitive deficits are associated with schizophrenia and show a progressive worsening, often being unresponsive to treatment. New antipsychotic molecules acting as antagonist at the serotoninergic 5-hydroxytryptamine receptor 7 (*e.g*. lurasidone) or partial agonists at dopamine D3 receptor (*e.g*. cariprazine) could have an impact on cognition in this patient group. The aim of the systematic review is to explore the efficacy of lurasidone and cariprazine in improving cognition in both animal models and human studies. The following terms: (lurasidone AND cognit*) OR (cariprazine AND cognit*) were searched in Web of Science from inception to December 2021. We included all studies that assessed changes in cognitive function after treatment with cariprazine or lurasidone. Of 201 selected articles, 36 were included. Twenty-four articles used animal models (rats, mice and marmosets), five evaluating the effects of cariprazine and 19 the effects of lurasidone. Twelve articles were clinical studies (cariprazine n = 2; lurasidone n = 10). In both animal and human studies lurasidone showed a greater efficacy on cognitive performance compared to placebo, quetiapine, ziprasidone or treatment-as-usual. Cariprazine was superior to other antipsychotics in improving cognitive functions in both animal and human studies. The cognitive effect of lurasidone could be explained by its potent antagonism at the 5-HT_7_ receptors combined with partial agonism at 5-HT_1A_ receptors. The pro-cognitive effect of cariprazine is probably explained by its very high affinity for D3 receptors. Head-to-head studies comparing lurasidone and cariprazine are needed to establish the “first-choice” treatment for cognitive dysfunction associated with schizophrenia.

## 1 INTRODUCTION

Schizophrenia is a severe psychiatric disorder, affecting more than 20 million people in the world. Schizophrenic patients show higher rates of morbidity and mortality (WHO, 2019) [1] and are subjected to the elevated social stigma which results in significant impairment in quality of life. Cognitive deficits are often associated with schizophrenia, even before its first onset, and show a progressive worsening after the diagnosis, thus representing a core feature of the disease [2, 3]. The biological mechanisms underlying cognitive impairments in schizophrenia seem to involve the cortico-cerebellar-thalamic-cortical pathways, including developmental abnormalities in both maturation and neurogenesis of neurons and synapses [4, 5]: specifically, grey matter alterations in the anterior cingulate are present even before the onset of the disease [6]. Despite the burden associated with cognitive impairment, the development of new medications for schizophrenia over the past decades provided only limited improvement in cognition, giving rise to the need for new pharmacological strategies [7, 8]. Recently, the introduction of antipsychotic molecules acting as an antagonist at the serotoninergic 5-hydroxytryptamine receptor 7 (*e.g*. lurasidone) or partial agonists at dopamine D3 receptor (*e.g*. cariprazine) could have an impact on cognitive outcomes in patients with psychotic disorders [9-11]. However, results from studies focusing on cognition after stimulation or blockade of these receptors are still controversial [12-15]. A potential explanation of the role of D3 receptor partial agonists or antagonists on cognition [16] may be the modulation of somatodendritic D3 receptors in the ventral tegmental area, leading to an increase in dopamine release in the prefrontal cortex [17]. On the other hand, antagonism of theserotonergic 5 HT-7 seems to reverse memory deficits through the regulation of hippocampal adenylyl cyclase activity [18].

### 1.1 Lurasidone

**Fig. (1):**
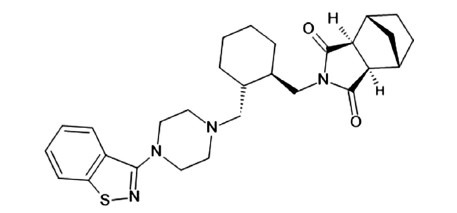
Structure of lurasidone.

**Fig. (2):**
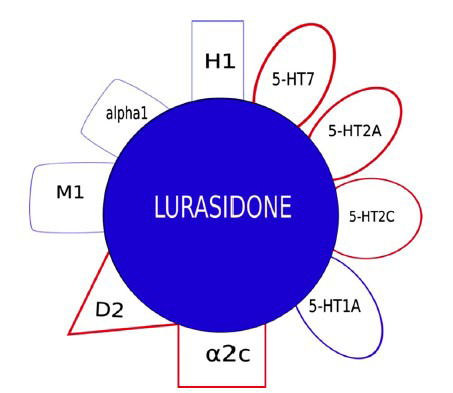
Pharmacological profile of lurasidone.

Lurasidone (Figs. **1** and **2**) is a potent antagonist of 5-HT_7_ receptors and a partial agonist of 5-HT_1A_ receptors. Both characteristics are predictive of the pro-cognitive and antidepressant activity of the drug [19, 20]. Lurasidone is also a high-affinity antagonist of 5-HT_2A_ and D2 receptors, and its use is associated with a low risk for extrapyramidal symptoms (EPS). The low affinity of lurasidone for M1 muscarinic, H1 histamine, 5-HT_2C_ and alpha1 adrenergic receptors suggests a low liability to cause peripheral and central anticholinergic adverse effects, sedation, weight gain and hypotension. Lurasidone is also an antagonist of α2c receptors, but the relevance of this mechanism in the overall pharmacological effect of the drug is unknown [19]. Dosing instructions recommend taking lurasidone once daily in the evening, to further reduce the incidence of EPS and sedation. A meal with a caloric value of at least 350 kcal, regardless of the fat content, is recommended to optimize lurasidone absorption. Lurasidone is rapidly absorbed from the gastrointestinal tract and reaches peak plasma concentrations within 1-3 hours. A steady state is reached after 7 days of administration with linear kinetics. Lurasidone has a very high plasma protein binding (99.8%), and is mainly metabolized in the liver by CYP3A4 through hydroxylation processes of the norbornene ring, with the formation of three active (ID-14283, ID-14326 and ID-14614) and two inactive (ID-20219 and ID-20220) metabolites present in percentage less than 10%. Lurasidone crosses the placental barrier and is excreted in the urine and feces. Dose adjustment is expected in patients with impaired renal clearance and severe hepatic insufficiency. Commonly adverse events are somnolence, akathisia, nausea, parkinsonism, and insomnia [21]. Lurasidone treatment is associated with a low risk of hyperprolactinemia, corrected QT (QTc) interval prolongation, weight gain and metabolic disturbances [22, 23]. Compared to olanzapine and quetiapine [24-26], lurasidone is associated with significantly less weight gain but higher rates of akathisia, anxiety and EPS. Lurasidone has a similar safety and tolerability profile to ziprasidone, except for a lower risk of somnolence and QTc prolongation [27].

### 1.2 Cariprazine

**Fig. (3):**
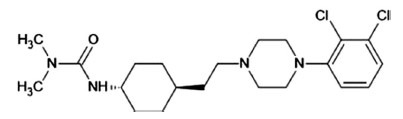
Structure of cariprazine.

**Fig. (4):**
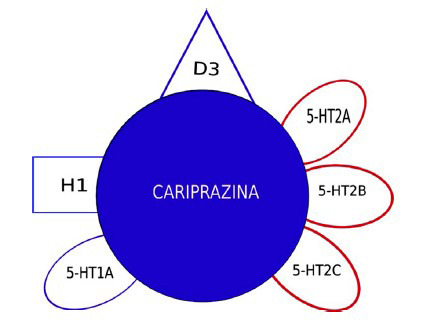
Pharmacological profile of cariprazine.

Cariprazine (Figs. **3** and **4**) shows a 10-fold higher affinity for human D3 versus human D2 receptors (binding affinity: pKi = 10.07 and 9.31, respectively), behaving as a partial agonist of both receptors. Cariprazine is also a partial agonist of 5-HT1A receptors (pKi = 8.59), a high affinity antagonist of 5-HT2B receptors (pKi = 9.24), and a low affinity antagonist of 5-HT2A (pKi = 7.73), H1 (pKi = 7.63), and 5-HT_2C_ (pKi = 6.87) receptors. By acting as a partial agonist at D3 and 5-HT_1A_ receptors, cariprazine improves negative symptoms, anhedonia and cognitive deficits associated with schizophrenia. Cariprazine has low affinity for adrenergic, and cholinergic receptors. The most common adverse events (≥ 10%) are akathisia, insomnia, weight gain and headache. The risk for cardiovascular adverse effects, sedation, hyperprolactinemia, and metabolic adverse effects is low [28, 29].

The time to reach peak plasma concentrations of cariprazine (Tmax) is approximately 3-8 hours. Cariprazine and its major active metabolites are strongly bound (92 to 97%) to plasma proteins. Cariprazine has two active metabolites, one of which has a half-life of 1-3 weeks. The functional total half-life is 7 days. Cariprazine is extensively metabolised by CYP3A4 and to a lesser extent by CYP2D6 to desmethyl cariprazine (DCAR) and didesmethyl cariprazine (DDCAR).

Cariprazine shows a consistent efficacy in improving the negative symptoms of schizophrenia. In a 26-week head-to-head study of cariprazine (3, 4.5, or 6 mg/d) *vs*. risperidone (3, 4, or 6 mg/d), cariprazine has shown greater efficacy in reducing blunted affect, emotional withdrawal, poor rapport, passive/apathetic social withdrawal, difficulty in abstract thinking, as well as total negative symptoms derived from the Positive and Negative Syndrome Scale (PANSS) of schizophrenia [30].

In another study [31], cariprazine was compared to risperidone in patients affected by schizophrenia with persistent and predominantly negative symptoms. Cariprazine had a greater efficacy on the PANSS negative factor score at week 26. This clinical trial also revealed a significant effect of cariprazine in the following items: self-care, socially useful activities as well as personal and social relationships.

### 1.3 Aim

Considering the potential influence of lurasidone and cariprazine on cognitive symptoms of schizophrenia, the aim of the present systematic review is to explore the efficacy of these two molecules in improving cognition in both animal models and human subjects.

## 2 MATERIALS AND METHODS

This systematic review was conducted according to the Preferred Reporting Items for Systematic Reviews and Meta-Analyses (PRISMA) guidelines [32]. We used the modified Cochrane Collaboration tool to assess the risk of bias for randomized controlled trials.

### 2.1 Search

The following search terms: (lurasidone AND cognit*) OR (cariprazine AND cognit*) were entered into the Web of Science research engine (all databases) from inception to December 2021. After duplicate removal, articles that were not relevant were excluded. Full-text version of the remaining articles was then obtained and screened according to the pre-specified eligibility criteria described below. Hand-searches of reference lists of potentially relevant articles, clinical studies, and reviews were conducted. The entire search process was conducted independently by three researchers, and disagreements at the final stage were resolved by consensus.

### 2.2 Inclusion/Exclusion Criteria

Inclusion criteria were: studies in English language that assessed changes in cognitive function in response to treatment with cariprazine or lurasidone. Studies were excluded if they did not report group comparisons or measurements of cognitive functions. Reviews, meta-analyses, conference proceedings/abstracts, book chapters, and unpublished theses were also excluded.

## 3 RESULTS

**Fig. (5):**
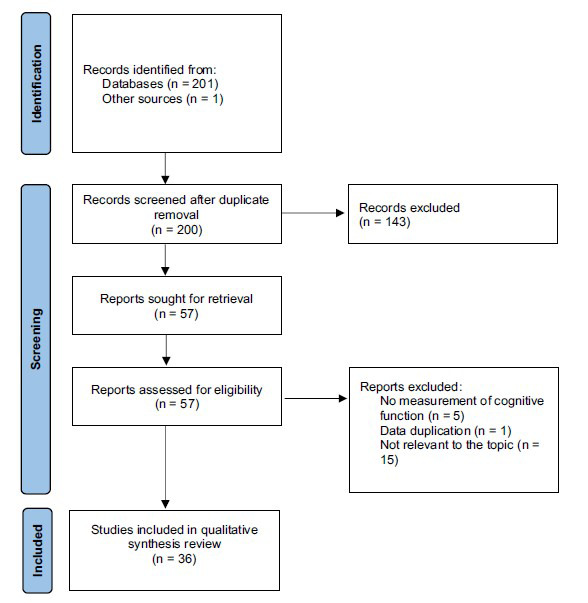
PRISMA flow-chart of the selected studies.

Of the 201 selected articles, 36 were included (Fig. **5**). Twenty-four were research articles using animal models (rats, mice and marmosets), five evaluating the effects of cariprazine and 19 effects of lurasidone. Twelve articles reported the results of clinical studies using cariprazine (n = 2) or lurasidone (n = 10).

### 3.1 Animal Studies

General characteristics of the selected studies are presented in Table **1**.

#### 3.1.1 Cariprazine

Five of the 25 identified studies evaluated the cognitive effects of cariprazine [33-37]. They all used the phencyclidine (PCP) model of schizophrenia, in which PCP treatment causes behavioural effects similar to negative symptoms in schizophrenia, as well as cognitive deficits involving long-term memory and executive memory. Barnes *et al.* 2018 [33] investigated the effect of systemic administration of cariprazine or aripiprazole in reversing memory deficits in a PCP model using the five-choice serial reaction time task (5-CSRTT). The 5-CSRTT is a behavioural test in which the animal must identify which of the five apertures has been briefly illuminated, *via* a nose poke, in order to receive a sugar reward. They found that all tested cariprazine doses (0.03, 0.1, or 0.3 mg/kg) significantly diminished the PCP-induced increases in incorrect, premature, and timeout responses. However, the two higher doses of cariprazine also reduced the number of completed trials, percent accuracy, and the number of correct responses, suggesting that these doses resulted in a non-specific suppression of responses. Aripiprazole treatment also attenuated PCP-induced deficits. Neill *et al.* 2016 [34] evaluated the effects of cariprazine in a PCP model using three different tasks: the Novel Object Recognition (NOR), the Operant Reversal Learning (ORL) and the Social Interaction (SI) tasks. The NOR is a commonly used behavioral assay for the investigation of various aspects of learning and memory in mice. During training, the mouse is allowed to explore 2 identical objects. On the test day, one of the training objects is replaced with a novel object. Because mice have an innate preference for novelty, if the mouse recognizes a familiar object, it will spend most of its time with the novel object (Lueptow *et al.* 2017) [38]. In the ORL test, rats are trained to press either a left or a right lever (only one is active) for food delivery, according to the presence or absence of a visual cue (LED light stimulus above lever). The social interaction test was performed in a square plexiglas open-field box. Two weight-matched rats (one treated test rat and one conspecific), unfamiliar with each other, were placed in the box together with an unfamiliar object (*e.g*., an unopened drink can). Behaviors (sniffing, avoidance and object exploration) are recorded. Neill and co-authors demonstrated that PCP-induced deficits in cognition and social behavior were significantly improved by cariprazine in a dose-dependent manner in the ORL test while showing efficacy at lower doses in the NOR and SI tests. Watson *et al.* (2016) [35] compared the effects of cariprazine and aripiprazole in the same PCP rat model to determine their ability to reverse behavioral changes using the NOR and the SI experimental conditions. Results suggest that cariprazine was as effective as aripiprazole in reversing deficits. Zimnisky *et al.* (2013) [36] tested the efficacy of a cariprazine pre-treatment in reducing the severity of cognitive deficits caused by PCP in wild-type or D3-receptor knockout mice. Cariprazine pre-treatment significantly attenuated the emergence of social recognition, spatial working memory, and attention deficits in PCP-treated wild-type mice, but not in PCP-treated D3-receptor knockout mice. This demonstrated the key role of D3-receptor in mediating the pro-cognitive effect of cariprazine. Gyertyan *et al.* (2011) [37] studied the effect of cariprazine in rats treated with scopolamine, a muscarinic receptor antagonist affecting learning and memory. Compared to aripiprazole, olanzapine and risperidone, cariprazine was the only antipsychotic agent capable of improving scopolamine-induced learning deficits.

#### 3.1.2 Lurasidone

Nineteen of the 24 identified studies evaluated the effects of lurasidone on cognition. Nine studies assessed the effects of lurasidone using the NOR test [39-48]. Calabrese *et al.*, (2020) [39] examined the effect of lurasidone treatment in rats exposed to chronic mild stress using the NOR test (see above for a description). Chronic mild stress produced an impairment of NOR performance by 30% which was completely reversed by lurasidone treatment. Interestingly, lurasidone did not influence cognitive performance in control rats. Using the same test, Horiguchi *et al.* (2011) [40] studied lurasidone, amisulpride, and sulpirid in a PCP model in rats. Lurasidone and amisulpride significantly attenuated the cognitive deficit induced by PCP and the effect of lurasidone was prevented by co-administration of a 5HT_7_ receptor agonist. This indicates that the pro-cognitive effect of lurasidone was mediated by 5-HT_7_ receptor blockade. In the same year, Horiguchi *et al.* (2011) [41] used the same model to evaluate the effect of a metabotropic glutamate2/3 (mGlu2/3) receptor agonist combined with either clozapine or lurasidone on cognition. Lurasidone combined with a mGlu2/3 receptor agonist could reverse the deficit in the NOR test, although it was inactive on its own. The same research group (Horiguchi *et al.*, 2012) [42] further examined the pro-cognitive effect of lurasidone in an attempt to clarify its mechanism(s) of action. If co-administered with PCP, lurasidone was able to prevent PCP-induced NOR deficit only at high doses. Of note, the effect of lurasidone was blocked by the co-administration of a 5-HT_1A_ receptor antagonist. The same effect was further confirmed in a subsequent experiment [43] in which the effect of lurasidone was similar to a postsynaptic 5-HT_1A_ agonist, F15599, in reversing PCP-induced deficit. Later, Horiguchi *et al.*, (2016) [44] demonstrated the efficacy of high doses of lurasidone in reversing PCP-induced NOR deficit for a longer period (14 days after the last lurasidone dose), as compared to other 5HT_1A_ agonists (*e.g*., tandospirone). A similar experiment was conducted by Huang *et al.*, (2018) [45], showing the relevance of 5HT_1A_ receptor activation (to increase the efflux of dopamine and acetylcholine in the cortex) and 5HT_7_ receptor blockade (involved in the modulation of glutamate efflux in the median prefrontal cortex). Another mechanism of action of lurasidone was studied by Miyauchi *et al.*, (2016) [46] in the same animal model. Specifically, they observed that nicotinic acetylcholine (ACh) receptor (nAChR) agonists (both α4β2* and α7nAChR agonists) could improve PCP-induced NOR deficit and potentiate the effect of sub-effective doses of lurasidone. More recently, the same group [47] observed that a combination of suboptimal doses of a D4 receptor agonist and lurasidone could improve cognition, suggesting that D4 receptors are also involved in the effect of lurasidone. Miyauchi, (2017b) [48] examined the contribution of M1 muscarinic receptors to the overall effect of atypical antipsychotics on cognitive fuction. Drugs with high affinity for M1 receptors, such as clozapine, reduced PCP-induced NOR deficit compared to lurasidone because of the M1 receptor antagonism. Another study [49] used the NOR, ORL, and SI tests to examine whether pregnenolone sulphate (PregS) alone or in combination with lurasidone could rescue persistent deficits in episodic memory, executive functioning, and social behaviour caused by subchronic treatment with PCP. A high dose of PregS significantly rescued subchronic PCP-induced NOR and SI deficits. The same effect was obtained by administering lower doses of PregS and lurasidone. Rajagopal *et al.* (2016) [50] used only the ORL test to examine the ability of 5-HT1A partial agonism and 5HT_7_ antagonism to improve cognition in PCP-treated mice. PCP significantly diminished the percent correct responding and increased the total incorrect trials and total incorrect responses in the reversal phase performance of the ORL test; pre-treatment with lurasidone reversed the ORL deficit in PCP-treated mice. The effect of lurasidone was reversed by a selective 5-HT_1A_ antagonist. Two studies assessed the cognitive effects of lurasidone using Object Retrieval with Detours (ORD) [51, 52]. Marmosets were trained to reach the reward (a piece of cake) set in a clear acrylic box open only at one side. The box was placed just outside the animal cage. Each test session consisted of 9 easy trials (reward placed within direct reach) and 8 difficult trials (reward placed deep within the box, and reaching it requires a detour around the box). The order of presentation was fixed and reaching the reward without touching any wall of the box within 30 s was considered “correct”. Marmosets were trained in one test session per day, 2 or 3 days a week. After the 14^th^ training session, animals were treated with antipsychotics and their performance in the ORD task was assessed. Murai *et al.* (2013) [51] showed that lurasidone did not affect the success rates in the easy trial of the task even at the highest dose while haloperidol, olanzapine, risperidone decreased the success rate; clozapine and quetiapine did not affect the success rate, but they caused adverse effects, such as drowsiness and emesis. Lurasidone increased the success rates also in the difficult trial, whereas haloperidol, olanzapine, quetiapine, risperidone, and clozapine decreased the success rate. Murai *et al.*, (2014) [52] examined the role of D4 receptors in the pro-cognitive effect of lurasidone on executive functions using selective D4 receptor agonists and antagonists. They observed that activation of D4 receptors may improve executive function, whereas D4 and D2 receptor blockade may have the opposite effect. Three studies used the passive avoidance (PA) test in rats treated with the NMDA receptor channel blocker, MK-801 [53-55]. The PA apparatus consisted of a lighted compartment and a dark compartment with a grid floor. These two compartments were separated by a sliding door. In the training session, the animals were placed in the lighted compartment and allowed to explore for 10 sec. The sliding door was then opened, and the step-through latency for animals to enter the dark compartment was measured. As soon as the animals entered the dark compartment, the door was closed: 3 sec later, an inescapable foot-shock was delivered through the grid floor. All animals examined entered the dark compartment within 300 sec as cut-off latency in the training session and received a foot-shock. The test session was performed 24 hours after the training session using the same paradigm, but without the foot-shock, and the step-through latency for animals to enter the dark compartment was measured. Horisawa *et al.* (2013) [53] showed that 5-HT_7_ receptor antagonism is involved in the pro-cognitive effects of lurasidone in MK-801-treated rats. Ishiyama *et al.* (2007) [54] showed that both pre-training and post-training administration of lurasidone significantly and dose-dependently reversed the impairment of the PA response. This may suggest that lurasidone worked, at least in part, by restoring the memory consolidation process disrupted by MK-801. Similar results were obtained by Kolaczkowski *et al.* (2014) [55]. Enomoto *et al.* (2008) [56] observed the reversal effect of lurasidone on MK-801-induced impairment of learning and memory using the Morris water maze (MWM) and radial-arm maze (RAM) tests in rats. Lastly, Percelay *et al.* 2020 [57] analyzed the tolerability of high doses of lurasidone in mice.

### 3.2 Humans

We identified 12 studies, performed in 7 countries, which met the inclusion criteria and for which the required data were available. Study characteristics are shown in Table **2**.

#### 3.2.1 Cariprazine

Fleischhacker *et al.* 2019 [30] evaluated the effect of cariprazine on cognitive function in schizophrenia, assessing changes in PANSS-derived cognitive factor scores. Cariprazine was superior to risperidone in reducing PANSS-based factors evaluating disorganized thoughts, prosocial function, and cognition even at week 26.

Vieta *et al.* 2021 [58] examined the effects of cariprazine on functional and cognitive outcomes in patients with bipolar I disorder, evaluating mean changes from baseline to week 8 in the Functional Assessment Short Test (FAST), a validated 24-item clinician-rated scale that was designed to measure the areas of functional difficulty associated with bipolar disorder [59]. Cariprazine was superior to placebo in reducing the FAST total score at week 8 at the dose of 1.5 mg/die. On the FAST subscales, cariprazine (1.5 mg/d) was better than placebo in terms of Autonomy, Occupational Functioning, Leisure Time, and Cognitive Functioning.

#### 3.2.2 Lurasidone

Three studies [60-62] assessed the effect of lurasidone using the CogState Computerized Cognitive Battery, a well-established assessment battery for cognitive impairment in schizophrenia [63]. Harvey *et al.* (2015) [60] performed a 6-month double blind trial of lurasidone compared to quetiapine. They observed schizophrenic patients who received either 120 or 160 mg/die of lurasidone showed significantly greater improvement in the overall cognitive performance compared to quetiapine extended release (XR) at a variable dose of 200-800 mg/die at week 32. Mean changes in neurocognitive composite z-score from baseline were significant for all lurasidone doses at both weeks 19 and 32. Functional capacity scores improved in all treatment groups. However, Miller *et al.* (2020) [61] did not observe a significant difference in cognitive performance in patients with schizophrenia randomized to lurasidone (80 or 160 mg/die) or quetiapine XR (600 mg/die) at 6 weeks from baseline. Lurasidone (160 mg/die) was superior to placebo in improving cognition and this effect was mediated by inflammation and metabolic risk measures (c-reactive protein levels, and triglyceride/HDL ratio) observed at baseline. In line with these findings, Raison *et al.* 2020 [62] performed a study in which patients (10 to 17 years of age) with bipolar I disorder were randomized to 6 weeks of double-blind treatment with once-daily, flexibly dosed lurasidone (20-80 mg) or placebo. They found that higher baseline levels of high-sensitivity c-reactive protein were associated with improvement in cognitive function in the lurasidone group (*vs.* placebo); this was observed however only in patents with normal BMI and not in overweight/ obese patients.

Two studies [64, 65] used the MATRICS Consensus Cognitive Battery (MCCB), a battery recommended by FDA to assess cognitive impairment in schizophrenia [66].

In Harvey *et al.* (2011) [64] adult outpatients who had schizophrenia or schizoaffective disorder were randomized to 21 days of double-blind treatment with lurasidone (120 mg once daily) or ziprasidone (80 mg twice daily). Lurasidone-treated patients showed a significant improvement from baseline on the MCCB composite score, while ziprasidone-treated patients did not show any improvement.

Kantrowitz *et al.* (2016) [65] conducted a multicenter, rater-blinded, randomized, controlled study of auditory-focused cognitive remediation used as an augmentation to lurasidone (40-160 mg/die). At study completion, there was a significant improvement in the MCCB composite score both in the auditory processing cognitive remediation and in the comparison group. It had to be noted that both groups were on open-label treatment with lurasidone, and this could have been the cause of the results.

Other three studies [67-69]; used the changes in PANSS-cognitive factors (as defined by Lindenmayer *et al.* 1994 [70]) as the outcome measures for cognition.

Harvey *et al.* (2017) [67] evaluated clinically unstable patients with schizophrenia, who were randomized to once-daily, fixed-dose treatment with lurasidone (80 or 160 mg), quetiapine XR (600 mg) or placebo, followed by a long-term, double-blind, flexible-dose continuation. Flexibly dosed lurasidone (40 to 160 mg/die) was found to be associated with significantly greater improvement in insight compared to flexibly dosed quetiapine XR (200 to 800 mg/d) over long-term treatment in patients with schizophrenia.

Nakamura *et al.* (2009) [68] performed a study in which patients were randomly assigned to 6 weeks of double-blind treatment with a fixed dose of lurasidone (80 mg) or placebo. They found that, at day 42, treatment with lurasidone was associated with significant improvement on the Brief Psychiatric Rating Scale as well as on the PANSS total score and the PANSS positive and general psychopathology subscales, including cognitive items. Significant improvement was seen as early as day 3.

Meltzer *et al.* (2020) [69] compared two doses of lurasidone (80 *vs*. 240 mg/die) in time to improve psychopathology and cognition during a 6-month trial in treatment-resistant schizophrenia patients. They found a significant non-dose-related improvement in the PANSS—total and subscales, and in 2 of 7 cognitive domains, *i.e*. speed of processing and executive function. In the same study, other types of cognitive evaluation focusing on executive functions, memory and attention showed a significant improvement from baseline only in the lurasidone 80 mg/die group.

Karpouzian-Rogers, Stocks *et al.*’s (2020) [71] study evaluated the effects of high versus low doses of lurasidone on Eye Movement performance in treatment-resistant schizophrenia. Eye movement measures are well-assessed paradigms used to examine the neural systems involved in cognitive and sensorimotor processes. In particular, the anti-saccade task is a reliable and sensitive measure of the processes involved in resolving the conflict between volitional and reflexive behavioral responses (Hutton *et al.* 2006) [72]. Patients completed the eye movement testing at baseline, on an existing medication regimen, after 6 weeks of low dose (80 mg) of lurasidone, after 12 weeks following randomization to low (80 mg) or high (240 mg) dose of lurasidone, and after 24 weeks of treatment. Six weeks of lurasidone treatment resulted in increased pro-saccade latency and reduced anti-saccade errors, with no change in memory-guided saccade accuracy. After randomization, saccade latencies increased only the high dose group, with no change in antisaccade errors in both groups. Memory-guided saccade error increased in the high-dose group and remained stable in the low-dose group, pointing to higher executive control in this group.

Lastly, Yatham *et al.* (2017) [73] aimed to examine the efficacy of lurasidone adjunctive therapy compared to treatment as usual in improving cognition in bipolar disorder type I, by administering the International Society for Bipolar Disorders Battery for Assessment of Neurocognition (ISBD-BANC). Lurasidone adjunctive therapy improved global cognition score. The effect of lurasidone on cognition score was of moderate to large magnitude.

## 4 DISCUSSION

Our systematic review shows the efficacy of lurasidone in improving cognition both in animal and human studies. According to animal models, the effect on cognition could be explained by its potent antagonism at the 5-HT_7_ receptors combined with partial agonism at 5-HT_1A_ receptors [74]. In fact, several animal studies [41, 42, 49, 50, 53, 55, 56] have shown that the pro-cognitive effect of lurasidone was mediated by either 5-HT_7_ receptor antagonism and/or 5-HT_1A_ receptor partial agonism. Functionally, the 5-HT_7_ receptor is associated with several physiological and pathological responses, including serotonin-induced phase shifting of the circadian rhythm, control of memory as well as locomotor and exploratory activity [75, 76]. Of note, two additional studies [46, 47] suggested that the action of lurasidone required the activation of D4 receptors. The D4 receptors are located presynaptically in glutamatergic terminals (thus potentially being involved in long-term potentiation and memory) while the postsynaptic localization is in the dendrites of the GABAergic efferent neurons [77]. The complex mechanism of action of lurasidone may be held responsible for its procognitive effect compared to other antipsychotic molecules which are also strong 5-HT_7_ antagonists as amisulpride. Of note, considering head-to-head confrontation in animal models, lurasidone was superior to haloperidol, olanzapine, risperidone, aripiprazole, clozapine and quetiapine in improving cognitive functions. We found more studies investigating the effect of lurasidone on cognition than the effect of cariprazine (29 *vs*. 7). In human studies, lurasidone showed a greater efficacy on cognitive performance compared to placebo [62, 68]; quetiapine [67, 60]; ziprasidone [64], or treatment as usual [72]. Only one study did not report a significant difference with quetiapine [61]. The positive effect on cognition was not consistently dose-dependent [69, 71]. However, the evidence pointing to a significant pro-cognitive effect of lurasidone in humans is still not very strong: in fact, one potential caveat of the included studies is that lurasidone was confronted with molecules (such as quetiapine) that have a higher sedation effect, which could, at least in part, biased the results. Comparisons with less sedating molecules such as aripiprazole are therefore needed.

According to animal models, the pro-cognitive effect of cariprazine is probably explained by its very high affinity for D3 receptors. The involvement of D3 receptors in the action of cariprazine was demonstrated using D3 receptor knockout mice, which were unresponsive to cariprazine. Cariprazine was superior to other antipsychotics in improving cognitive functions in animal studies [33, 34, 37]. In humans, there are little data to draw reliable conclusions: in fact, cariprazine was superior to placebo in one study and risperidone in the other; the same caveat observed for lurasidone is also valid for cariprazine studies, as risperidone has a higher sedation potential compared to cariprazine and this effect could have impact cognitive performance. One potential limitation of our study was the search in only one public database: this may have reduced the original number of articles included, but it is improbable that it may have affected the number of included studies that were thoroughly hand-searched for additional references.

## CONCLUSION

In conclusion, our data may support the use of lurasidone which appears effective on the psychopathological front as other “older” antipsychotics, but seems to have a more promising profile on cognition. Therefore, its use could be more frequent in younger patients or first-episode, in order to maintain the cognitive capacity of the individual. There is insufficient evidence for cariprazine, even if its use may seem potentially relevant. Head-to-head studies comparing lurasidone and cariprazine are awaited to establish which of the two drugs may be considered firstly in the treatment of cognitive dysfunction associated with schizophrenia.

## Figures and Tables

**Table 1:** General characteristics of animal studies.

**Author/** **Year**	**Country**	**Study Design**	**Trial Duration**	**Population**	**N**	**Induced Deficits Intervention**	**Intervention with Lurasidone or Cariprazine**	**Other Interventions (Placebo ** **or Other Antipsychotics)**	**Assessment**	**Cognition Findings**
Barnes *et al.* (2018) [33]	USA, Hungary	RCT	9 days	Male Wistar rats	96	PCP 2 mg/kg s.c.	Cariprazine 0.03, 0.1, or 0.3 mg/kg p.o.	2% Tween80 p.o.; Aripiprazole 1, 3, or 10 mg/kg p.o.	5-CSRTT	Cariprazine demonstrated potential in attenuating PCP-induced deficits in the 5-CSRTT performance.
Calabrese *et al.* (2020) [39]	Italy, Poland	RCT	7 weeks	Male Wistar rats	//	CMS (chronic mild stress)	Lurasidone 3 mg/kg p.o.	1% hydroxyethylcellulose p.o.	NOR	Seven weeks of CMS induced anhedonia and cognitive impairment, which were normalised by lurasidone.
Enomoto *et al.* (2008) [56]	Japan	RCT	5 days	Male Wistar rats	//	MK-801 0.15 and 0.2 mg/kg i.p.	Lurasidone 1 and 3 mg/kg p.o.	Risperidone 0.3 and 1 mg/kg p.o.; Clozapine 3 and 10 mg/kg p.o.; Aripiprazole 0.3 and 1 mg/kg p.o.; Haloperidol 0.3 and 1 mg/kg p.o.	MWM and RAM	Lurasidone potently reversed MK-801-induced learning impairment in the MWM test and reference memory impairment in the RAM test.
Gyertyan *et al.* (2011) [37]	Hungary	RCT	//	Male Wistar rats	//	PCP 2 mg/kg s.c., MK-801 0.1 mg/kg i.p.	Cariprazine 0.01-2 mg/kg p.o.	Aripiprazole 2-80 mg/kg p.o.; olanzapine 0.3-3 mg/kg p.o.; risperidone 0.05-0.5 mg/kg p.o.	Water-labyrinth learning performance	Cariprazine 0.02-0.08 mg/kg significantly improved the learning performance of scopolamine-treated rats in a water-labyrinth learning paradigm. Risperidone, olanzapine, and aripiprazole were less active against phencyclidine and more cataleptogenic than cariprazine, and had no significant effect in the learning task.
Horiguchi *et al.* (2012) [42]	USA, Japan	RCT	22 days	Female Long-Evans rats	103	PCP 2 mg/kg bid, i.p.	Lurasidone 0.1 mg/kg or 1 mg/kg i.p.	Tandospirone 5 mg/kg i.p.; pimavanserin 3 mg/kg i.p.; haloperidol 1 mg/kg i.p.; WAY100635 0.6 mg/kg, saline	NOR	Lurasidone (1 mg/kg) but not 0.1 mg/kg, which is effective to acutely reverse the deficit due to subchronic PCP, or tandospirone, but not pimavanserin or haloperidol, significantly prevented the PCP-induced NOR deficit on day 15. The ability of lurasidone co-treatment to prevent the PCP-induced NOR deficit was enduring and still present at day 22. The preventive effect of lurasidone was blocked by WAY100635, a selective 5-HT1A antagonists, further evidence for the importance of 5-HT1A receptor stimulation in the NOR deficit produced by subchronic PCP.
Horiguchi *et al.* (2011) [40]	USA, Japan	RCT	> 1 month	Female Long-Evans rats	34	PCP 2 mg/kg bid, i.p.	Lurasidone 0.01 mg/kg or 0.03 mg/kg, 0.1 mg/kg, 0.5 mg/kg i.p.	Clozapine 0.1-0.3 mg/kg, LY379268 1-3 mg/kg, LY341495 1 mg/kg, haloperidol 0.1 mg/kg, pimavanserin 3 mg/kg, saline	NOR	mGlu2/3 agonism is relevant to the ability of clozapine and lurasidone to ameliorate the effect of subchronic PCP treatment on NOR, a putative model for the cognitive dysfunction of schizophrenia.
Horiguchi *et al.* (2011) [41]	USA, Japan	RCT	> 1 month	Female Long-Evans rats	129	PCP 2 mg/kg bid i.p	Lurasidone 0.03 and 0.1 mg/kg i.p.	Pimavanserin 3 mg/kg i.p., haloperidol 0.03 mg/kg i.p.; sulpiride 20 and 30 mg/kg i.p.; amisulpride 1 mg/kg i.p.; AS19 5 and 10 mg/kg i.p.; LY379268 1 mg/kg i.p; LY341495 1 mg/kg i.p.; SB269970 0.1, 0.3, and 1 mg/kg i.p.; saline	NOR	Lurasidone (0.1 mg/kg) significantly attenuated the PCP-induced deficit. Lurasidone (0.03 mg/kg) plus 0.1 mg/kg SB269970, but not 0.03 mg/kg lurasidone alone, significantly reversed the PCP-induced NOR deficit.
Horiguchi *et al.* (2012b) [43]	USA	RCT	> 1 month	Female Long-Evans rats	86	PCP 2 mg/kg bid i.p.	Lurasidone 0.1 mg/kg i.p.	Tandospirone 0.2 and 0.6 mg/kg i.p.; F15599 0.16 mg/kg i.p.; WAY100635 0.6 mg/kg i.p.; pimavanserin 3 mg/kg i.p.; buspirone 1 mg/kg i.p.; haloperidol 0.1 mg/kg i.p.	NOR	Lurasidone (0.1 mg/kg) and tandospirone (0.6 mg/kg) ameliorated the subchronic PCP-induced-NOR deficit. The combination of sub-effective doses of tandospirone (0.2 mg/kg) and lurasidone (0.03 mg/kg) also reversed the PCP-induced NOR-deficit.
Horiguchi *et al.* (2016) [44]	USA, Japan	RCT	5 weeks	Female Long-Evans rats	102	PCP 2 mg/kg bid i.p.	Lurasidone 0.1 and 1 mg/kg i.p.	Tandospirone 0.6 and 5 mg/kg i.p.; WAY100635 0.6 mg/kg i.p.; saline	NOR	Subchronic lurasidone (1, but not 0.1 mg/kg) significantly reversed the PCP-induced NOR deficit at 24 h after the last injection. The effect of lurasidone persisted for one more week.
Horisawa *et al.* (2013) [53]	Japan	RCT	//	Male Wistar rats	//	MK-801 0.075 mg/kg, s.c.	Lurasidone 3 mg/kg, p.o.	AS19 1, 3 and 10 mg/kg, s.c.	PA	The 5-HT_7_ receptor antagonistic activity of lurasidone plays an important role in its effectiveness against MK-801-induced deficits and may contribute to its pharmacological actions in patients with schizophrenia.
Huang *et al.* (2018) [45]	USA, Republic of Korea	RCT	> 2 weeks	Male wild-type C57BL/6J mice and 5-HT7RKO (constitutive KO) mice	//	PCP 10 mg/kg i.p., 5HT7-KO	Lurasidone i.p.	AS 19 10 mg/kg i.p.; WAY-100635 0.6 mg/kg i.p.; SB-269970 3 mg/kg i.p., saline	NOR	Acute lurasidone treatment reversed the cognitive deficit in NOR in subchronic PCP-treated mice.
Ishiyama *et al.* (2007) [54]	Japan	RCT	//	Male Wistar rats	//	MK-801 0.05 mg/kg, s.c.	Lurasidone 1-30 mg/kg p.o	Quetiapine 1-30 mg/kg p.o.; Haloperidol 0.3 and 1 mg/kg p.o.; Risperidone 0.3-3 mg/kg; Olanzapine 0.3-10 mg/kg; Aripiprazole 0,3-10 mg/kg; Clozapine 0.3-30 mg/kg p.o.	PA	Lurasidone is superior to other antipsychotics in improving the MK-801-induced memory impairment.
Kolaczkowski *et al.* (2014) [55]	Poland, France	RCT	//	Male Wistar rats	7-8 per group	MK-801 0.3 mg/kg i.p.	Lurasidone 0.3 to >100 i.p	Aripiprazole 10-100 mg/kg i.p., Olanzapine 0.3-10 mg/kg i.p., risperidone 0.1-10 mg/kg i.p., lurasidone 1-100 mg/kg i.p., asenapine 0.03-10 mg/kg i.p., clozapine 1-100 mg/kg i.p., chlorpromazine 1-30 mg/kg i.p., haloperidol 0.01-0.3 mg/kg s.c., imipramine 3-10 mg/kg	PA	Lurasidone dose-dependently and significantly antagonized hyperlocomotion, reduced immobility time in the forced swimming test, reduced the step-through latency in the passive avoidance test, inhibited spontaneous locomotion and did not elicit catalepsy.
Miyauchi *et al.* (2016) [46]	USA, Japan	RCT	>1 month	Female Long-Evans rats	204	PCP 2mg/kg bid i.p.	Lurasidone 0.03-0.3 mg/kg i.p.	A-83580 0.3 mg/kg i.p., DHbetaE 3 mg/kg i.p., PNU282987 1 mg/kg i.p., MLA 1 mg/kg i.p., MEC 1 mg/kg i.p.	NOR	The increase of hippocampal ACh efflux by lurasidone may contribute to its efficacy to restore NOR in the scPCP model.
Miyauchi *et al.* (2017) [47]	USA, Japan	RCT	> 1 month	Female Long-Evans rats	223	PCP 2mg/kg bid i.p.	Lurasidone 0.03-0.1 mg/kg	L-745,870 1 and 3 mg/kg i.p.; PD168077 0.5, 1.5 and 3 mg/kg i.p.; clozapine 0.1 and 0.3mg/kg i.p.; saline	NOR	A SED (subeffective dose) of the D4 agonist, PD168077 (0.5 mg/kg), potentiated the ability of a SED of the atypical APD, lurasidone (0.03 mg/kg), to reverse the sub-chronic PCP-induced NOR deficit, while only partially potentiating a SED of clozapine (0.1 mg/kg). Lastly, D4 antagonism with L-745,870 (1 mg/kg), at a dose that does not disrupt NOR in saline-treated animals, blocked the ability of clozapine (0.3 mg/kg), but not lurasidone (0.1 mg/kg), to reverse the sub-chronic PCP-induced NOR deficit.
Miyauchi *et al.* (2017) [48]	USA, Japan	RCT	> 1 month	Female Long-Evans rats	228	PCP 2mg/kg bid i.p.	Lurasidone 0.03-0.1 mg/kg	Scopolamine 0.03 and 0.1 mg/kg i.p.; VU0255035 3 and 10 mg/kg i.p.; AC260584 1 and 3 mg/kg i.p.; NDMC 0.3 and 3 mg/kg i.p.; clozapine 0.1 and 0.3 mg/kg i.p.; saline	NOR	mAChR antagonism, specifically of the M1 receptor, preferentially disrupts the ability of clozapine and NDMC to fully rescue the subchronic PCP-induced NOR deficit compared to lurasidone; co-administration of the M1 agonist, AC260584, potentiates the effects of NDMC (0.3 mg/kg), clozapine (0.1 mg/kg), and lurasidone (0.03 mg/kg) to reverse the subchronic PCP-induced deficit in NOR.
Rajagopal *et al.* (2013) [49]	Japan	RCT	//	Male and female common marmosets	105	//	Lurasidone 0.3, 1, 3, 10 mg/kg into the stomach	Haloperidol 0-1 mg/kg, olanzapine 0-10 mg/kg, risperidone 0-1 mg/kg, quetiapine 0-30 mg/kg, clozapine 0-10 mg/kg (all drugs into the stomach)	ORD	Lurasidone, unlike conventional antipsychotics, improves cognition associated with executive function.
Murai *et al.* (2014) [52]	Japan	RCT	//	Male and female common marmosets	23	//	Lurasidone 0-10 mg/kg into the stomach	Clozapine 0-10 mg/kg, L-745970 0-10 mg/kg, Ro10-5824 0-3 mg/kg (all drugs into the stomach)	ORD	The lack of affinity for the dopamine D4 receptor by lurasidone could contribute to its cognitive-enhancing effect.
Neill *et al.* (2016) [34]	UK, Hungary, USA	RCT	> 2 weeks	Female Lister Hooded rats	240	PCP 2 mg/kg i.p.	Cariprazine 0.05, 0.1, or 0.25 mg/kg, p.o.	Risperidone 0.16 mg/kg i.p.	NOR, ORL and SI	Cariprazine was effective to overcome PCP-induced deficits in cognition and social behavior.
Percelay *et al.* (2020) [57]	France	RCT	5 weeks	Male C57BL/6J mice	20	//	Lurasidone 1 mg/kg i.p., 8.3 mg/kg p.o.	Saline	Behavioural experiment: Open field, spontaneous alternation, sociability and social recognition, NOR, PPI	An impact of chronic lurasidone administration in C57BL/6 mice behaviour wasn't observed.
Rajagopal *et al.* (2016) [50]	USA	RCT	> 2 weeks	Male C57BL/6J mice	96	PCP 10 mg/kg i.p.	Lurasidone 1 or 3 mg/kg; i.p	Tandospirone 0.1, 1, or 5 mg/kg i.p.; WAY100635 0.6 mg/kg i.p.; SB269970 0.5, 1, or 4 mg/kg i.p.; AS 19 10 mg/kg i.p.	ORL	Lurasidone reversed ORL deficit in the scPCP-treated mice.
Rajagopal *et al.* (2018) [49]	USA	RCT	>1 week	Male C57BL/6J mice	//	PCP 10 mg/kg i.p.	Lurasidone 0.1 mg/kg i.p.	PregS 3-30 mg/kg i.p., Tandospirone 0.03-1 mg/kg i.p., saline, 0.05% methylcellulose and 0.2% Tween80	NOR, ORL, SI	Acute lurasidone administration rescued scPCP-induced cognitive deficits in male C57BL/6 J mice.
Watson *et al.* (2016) [35]	UK, Hungary, USA	RCT	> 5 weeks	Male Lister hooded rats	12-16	PCP 10 mg/kg i.p.	Cariprazine 0.03-0.3mg/kg i.p.	aripiprazole 0.3-3mg/kg i.p.	NOR	Cariprazine is at least as effective as aripiprazole and in some paradigms, it showed additional beneficial features.
Zimnisky *et al.* (2013) [36]	USA, Hungary	RCT	//	C57BL/6J mice	//	PCP 1 mg/kg i.p., D3-receptor KO	Cariprazine 0.005 to 0.15 mg/kg i.p.	//	EPM, SI, Recognition Memory, WM, ASST	Cariprazine pretreatment significantly attenuated the emergence of cognitive deficits in PCP-treated wild-type mice, but not in PCP-treated D3-receptor knockout mice.

**Abbreviations**: ASST (Attention-Set-Shifting Task), CAR (Conditioned avoidance response), CIAS (Cognitive Impairment Associated with Schizophrenia), EPM (Elevated plus maze), FST (Forced swim test), MWM (Morris water maze), NOR (Novel Object Recognition), ORD (Object Retrieval Detour), ORL (Operant Reversal learning), PA (Passive avoidance), PCP (Phencyclidine), PPI (Prepulse inhibition), RCT (Randomized Clinical Trial), RAM (Radial-arm maze), SI (Social interaction), WM (Working memory), 5-CSRTT (Five-choice serial-reaction time task).

**Table 2:** General characteristics of human studies.

**Author/** **Year**	**Country**	**Study Design**	**Trial Duration**	**Population**	**N Tot**	**N**	**N ** **Ctrl**	**Diagnosis**	**Diagnostic Criteria**	**Intervention**	**Intervention Ctrl**	**Assessment**	**Cognition ** **Findings**
Fleischhacker *et al.* (2019) [30]	Austria, Italy, USA, Hungary	Double blind RCT	26 weeks	Male and female patients (18-65 years old)	454	227	227	Schizophrenia	DSM-IV-TR	Cariprazine 3, 4.5, or 6 mg/d	Risperidone 3, 4, or 6 mg/d	PANSS/PANSS derived factors	Changes from baseline was significantly different for cariprazine versus risperidone on PANSS-based factors evaluating cognition; (*P* < .05).
Harvey *et al.* (2011) [64]	USA, Japan	Double blind RCT	3 weeks	Male and female outpatients (18-70 years old)	301	150	151	Schizophrenia or Schizoaffective disorder	DSM-IV	Lurasidone 120 mg/d	Ziprasidone 80 mg BID	MCCB and SCoRS	Lurasidone patients demonstrated significant within group-improvement from baseline on the MCCB composite score (*p* = 0.026) and on the SCoRS (*p* < 0.001).
Harvey *et al.* (2015) [60]	USA, Hong Kong, Japan	Double-blind RCT	32 weeks	//	488	246	116; 120	Schizophrenia	DSM-IV-TR	Lurasidone 40-160 mg/d	Quetiapine XR 200-800 mg/d; Placebo	UPSA-B	All doses of lurasidone were superior to all doses of quetiapine for cognitive performance.
Harvey *et al.* (2017) [67]	USA	Double-blind RCT	6 months	//	292	151	85; 56	Acute exacerbation of Schizophrenia	DSM-IV-TR	Lurasidone 40-160 mg/d	Quetiapine XR 200-800 mg/d; Placebo	PANSS, UPSA-B and QWB-SA	At Week 32, improvement in PANSS total score was significantly greater in the lurasidone group compared to the quetiapine XR group. Treatment-related improvement in “insight and judgment” (PANSS-item G12 score) from acute phase baseline to Week 32 was significantly associated with improvement in neurocognitive performance.
Kantrowitz *et al.* (2016) [65]	USA	RCT	6 months	Male and female patients (18-55 years old)	120	120	//	Schizophrenia	DSM-IV-TR	Lurasidone 40-160 mg/d	//	MCCB and UPSA-B	Moderate effect size improvements were seen across group for cognitive and symptom outcomes, although no statistically significant between-group differences were seen at study completion.
Karpouzian-Rogers *et al.* (2020) [71]	USA	RCT	24 weeks	Male and female patients (18-60 years old)	43	21	22	Treatment-resistant schizophrenia	DSM-IV	Lurasidone 80 mg/d	Lurasidone 240 mg/d	EM (eye movement) measures	Stabilization on low dose lurasidone was associated with improved executive control of attention reflected by reduced antisaccade errors. High dose lurasidone resulted in prolonged speed of reflexive and executive shifts of attention and reduced spatial working memory relative to low dose.
Meltzer *et al.* (2020) [69]	USA	Double blind RCT	24 weeks	Male and female outpatients	67	34	33	Treatment-resistant schizophrenia	DSM-IV	Lurasidone 80 mg/d	Lurasidone 240 mg/d	PANSS, CGI, PSP, WCST, WISC-R Mazes, Letter Fluency, Category Fluency: Animal Naming, the Brown-Peterson Auditory Consonant Trigrams Test, DSST, Rey Auditory Verbal Learning Test.	Significant non-dose-related improvement in the Positive and Negative Syndrome Scale—Total and subscales and in 2 of 7 cognitive domains, speed of processing and executive function, were noted.
Miller *et al.* (2020) [61]	USA, Hong Kong	Double blind RCT	6 weeks	Male and female patients (18-75 years old)	488	125; 121	120;122	Schizophrenia	DSM-IV-TR	Lurasidone 80 and 160 mg/d	Quetiapine XR 600 mg/day; placebo	CRP and CogState	Baseline CRP level combined with measures of metabolic risk significantly moderated the improvement in cognitive performance associated with lurasidone 160 mg/day (*vs*. placebo) treatment.
Nakamura *et al.* (2009) [68]	Japan	Double blind case-control study	6 weeks	Male and female patients (18-64 years old)	180	90	90	Acute exacerbation of Schizophrenia	DSM-IV	Lurasidone 80 mg/d	Placebo	BPRSd, PANSS, CGI-S, MADRS	Treatment with lurasidone was associated with significant improvement compared to placebo on the BPRSd, as well as on all secondary efficacy measures, including the PANSS total score, PANSS positive, negativeand general psychopathology subscales.
Raison *et al.* (2020) [62]	USA, Hong Kong	Double blind case-control study	6 weeks	Male and female patients (10-17 years old)	343	173	170	Patients 10-17 years of age with a DSM-5 diagnosis of bipolar I depression	DSM-5	Lurasidone 20-80 mg/d	Placebo	CDRS-R, computerized Brief Cogstate Battery	Young patients with bipolar depression with normal weight and higher levels of pre-treatment CRP may show a greater placebo-adjusted improvement in depressive symptoms and cognitive performance when treated with lurasidone.
Vieta *et al.* (2021) [58]	Spain, USA	Randomized, double-blind, placebo-controlled, parallel-group, fixed-dose study	8 weeks	Male and female patients (18-65 years old)	393	135; 126	132	Bipolar I disorder without psychotic features	DSM-IV-TR	Cariprazine 1,5 or 3 mg/d	Placebo	FAST	The least squares mean difference (LSMD) in FAST total score change from baseline to week 8 was statically significant in favor of cariprazine 1.5 mg/d versus placebo (*p* = .0051); the LSMD versus placebo was statistically significant in favor of cariprazine 1.5 mg/d for Autonomy, Occupational Functioning, Cognitive Functioning, and Leisure Time.
Yatham *et al.* (2017) [73]	Canada	Randomized open-label pilot study	6 weeks	Male and female patients (19-65 years old)	34	17	17	Euthymic bipolar I disorder	DSM-IV-TR	Lurasidone 20-80 mg/d	TAU	YMRS, MADRS, CGI-BP, CFQ, BFIS, SDS, QoL.BD, ISBD-BANC, CVLT-II and BVMT-R	The magnitude of benefit with lurasidone adjunctive therapy in improving global cognition (effect size 0·46) was greater compared with the improvement observed in the TAU group (0·04).

**Abbreviations**: PANSS (Positive and Negative Symtomps Scale), MCCB (Matrics Consensus Cognitive Battery), SCoRS (Schizophrenia cognition rating scale), UPSA-B (The University of California San Diego (UCSD) Performance-based Skills Assessment-Brief version), QWB-SA (Quality of well-being self-administered), CGI (Clinical global impression), PSP (Personal and Social Performance Scale), WCST (Wisconsin card sorting test), WISC-R (Wechsler Intelligence scale for children), DSST (Digit symbol substitution test), CRP (C-Reactive Protein), BPRS (Brief psychiatric rating scale), MADRS (Montgomery Asberg Depression rating scale), CDRS (Clinical dementia rating scale), YMRS (Young mania rating scale), CFQ (Cognitive failure questionnaire), BFIS (Barkley functional impairment scale), SDS (Sheehan Disability Scale), QoL.BD (Quality of Life in Bipolar Disorder), ISBD-BANC (International Society for Bipolar Disorders-Battery for Assessment of Neurocognition), CVLT-II (California Verbal Learning Test–II), BVMT-R (Brief Visuospatial Memory Test—Revised version), TAU (treatment as usual).

## LIST OF ABBREVIATIONS

EPS: Extrapyramidal Symptoms
FAST: Functional Assessment Short Test
NOR: Novel Object Recognition
ORL: Operant Reversal Learning
PA: Passive Avoidance
PANSS: Positive and Negative Syndrome Scale
PRISMA: Preferred Reporting Items for Systematic Reviews and Meta-Analyses
SI: Social Interaction
